# Sports and dietary behaviour among children and adolescents in Germany. Results of the cross-sectional KiGGS Wave 2 study and trends

**DOI:** 10.17886/RKI-GBE-2018-070

**Published:** 2018-06-27

**Authors:** Susanne Krug, Jonas D. Finger, Cornelia Lange, Almut Richter, Gert B. M. Mensink

**Affiliations:** Robert Koch Institute, Department of Epidemiology and Health Monitoring

**Keywords:** SPORTS PARTICIPATION, FOOD CONSUMPTION, INFLUENCING FACTORS, KIGGS, HEALTH MONITORING

## Abstract

This article focuses on selected indicators related to sports and dietary behaviour – two important factors that influence the development of obesity. The analyses are based on data collected for the second wave of the German Health Interview and Examination Survey for Children and Adolescents (KiGGS Wave 2), which was conducted between 2014 and 2017. These data were collected, using a questionnaire, from 6,810 girls and 6,758 boys aged between 3 and 17. The analyses also compare the data collected for wave 2 with those from the KiGGS baseline study (2003-2006).

More than 70% of 3- to 17-year-olds state that they participated in sports. However, boys do so significantly more often than girls, and 11- to 17-year-olds do so more frequently than 3- to 10-year-olds. In addition, there is a correlation between children’s and adolescents’ sports participation and those of their parents, and with an activity-friendly living environment.

Younger children and girls have healthier diets than older children and boys. However, although the consumption of confectionery and sugary drinks by 3- to 17-year-olds has declined significantly since the KiGGS baseline study was conducted, 11- to 17-year-olds, in particular, eat significantly smaller amounts of vegetables than they did about ten years ago. Significantly more 3- to 10-year-olds currently eat at least five servings of fruit and vegetables per day than ten years ago, although the proportion of the children who reach this recommendation continues to remain very low at 14% overall.

It is important to set an example by following a healthy lifestyle within families and other settings in early life. Furthermore, the living environments also need to be made more health-oriented to support children and adolescents in reaching the national recommendations on physical activity and healthy eating.

**KiGGS Wave 2**

Second follow-up to the German Health Interview and Examination Survey for Children and Adolescents

**Data owner:** Robert Koch Institute

**Aim:** Providing reliable information on health status, health-related behaviour, living conditions, protective and risk factors, and health care among children, adolescents and young adults living in Germany, with the possibility of trend and longitudinal analyses

**Study design:** Combined cross-sectional and cohort study

**Cross-sectional study in KiGGS Wave 2**

**Age range:** 0-17 years

**Population:** Children and adolescents with permanent residence in Germany

**Sampling:** Samples from official residency registries - randomly selected children and adolescents from the 167 cities and municipalities covered by the KiGGS baseline study

**Sample size:** 15,023 participants

**KiGGS cohort study in KiGGS Wave 2**

**Age range:** 10-31 years

**Sampling:** Re-invitation of everyone who took part in the KiGGS baseline study and who was willing to participate in a follow-up

**Sample size:** 10,853 participants

**KiGGS survey waves**

► KiGGS baseline study (2003-2006), examination and interview survey
► KiGGS Wave 1 (2009-2012), interview survey
► KiGGS Wave 2 (2014-2017), examination and interview survey

More information is available at www.kiggs-studie.de/english

## 1. Introduction

The prevalence (frequency) of obesity has increased significantly throughout the world over the last few decades, and it is now a key issue in health promotion and disease prevention [1]. Current results from the second wave of the German Health Interview and Examination Survey for Children and Adolescents (KiGGS Wave 2), which was conducted between 2014 and 2017, show that 15.4% of 3- to 17-year-old children and adolescents in Germany are overweight, and that almost 6% are obese [2]. Although a comparison with data from the KiGGS baseline study (2003-2006) shows that the prevalence of overweight and obesity among children and adolescents has not increased over the last ten years, these conditions have continued to stagnate at a high level [2]. Childhood obesity is associated with a risk of long-lasting obesity that can continue into adulthood [3]. Moreover, the progression of obesity in adulthood is often even more severe in cases where the condition developed in childhood or adolescence [4]. Obesity is also associated with conditions such as cardiovascular diseases, type 2 diabetes as well as the metabolic syndrome, which reflects the co-occurrence of multiple cardiovascular risk factors.

Alongside genetics [5], the main cause of obesity is a permanent imbalance between energy intake and energy use [6]. Unhealthy eating habits, sedentary activities and a lack of physical activity, therefore, are important factors that influence obesity [7, 8]. It is important to note that even children and adolescents currently spend most of their day doing sedentary activities in school, as part of training or in the workplace. Furthermore, media-related related activities during leisure time can contribute towards further periods of physical inactivity. Sedentary behaviour has established itself in recent years as an independent risk factor for obesity. In 2010, the World Health Organization (WHO) defined minimum recommendations for physical activity in childhood and adolescence [9], and according to this, children and adolescents should undertake at least 60 minutes of moderate to vigorous physical activity every day. Currently, only 25.9% of children and adolescents meet these recommendations [10], and these only represent the minimum recommended levels. Current national guidelines for Germany go even further, and recommend at least 180 minutes for children aged between four and six, and at least 90 minutes daily for children between six and eleven to achieve greater health benefits [11].

At the same time, food tends to be constantly available almost everywhere. Access to energy-rich foods is often particularly easy, and this causes them to be consumed in large amounts, resulting in increased energy intake. A further risk factor for obesity in childhood and adolescence are the food products that particularly appeal to children, as these are often potentially unhealthy [12]. Further risk factors are the consumption of large amounts of sugary drinks [13, 14] and a low dietary intake of fruit and vegetables [15]. During the Eating Study as a KiGGS Module (EsKiMo), which was a module of the KiGGS baseline study, the results showed that children and adolescents often did not meet the recommendations for a healthy diet [16].

An individual’s understanding of health and health-related behaviour are influenced by various obesity-promoting (obesogenic) environmental factors [17-19]. The living environments of children and adolescents need to be made more health-oriented. The lack of safe cycle lanes and paths for pedestrians as well as poor accessibility to activity-friendly places foster a predominantly physical inactive lifestyle and consequently less energy expenditure [20]. On the other hand, the abundance of food stores and the supply of unhealthy products lead to increased energy intake.

For children and adolescents in Germany, current representative data from KiGGS Wave 2 are available on sport- and dietary behaviour as well as on obesity-promoting environmental factors. This article analyses the current sports and dietary behaviour of 3- to 17-year-old children and adolescents in Germany using selected indicators as well as environmental factors that favour obesogenic behaviour. In addition, in order to determine developments over time, these data from children and adolescents are compared to data from children of the same age that were collected about ten years ago.

## 2. Methodology

### 2.1 Study design

KiGGS is part of the health monitoring system at the Robert Koch Institute. The KiGGS study involves repeatedly performed representative cross-sectional surveys of children and adolescents aged between 0 and 17 in Germany (KiGGS cross-sectional component). The KiGGS baseline study was carried out as an examination and interview survey (2003-2006) while KiGGS Wave 1 was an interview-based survey (2009-2012). KiGGS Wave 2 (2014-2017) took place as a combined examination and interview survey. The KiGGS data were collected within a complex two-step sampling design that resulted in a clustered, stratified sample. Sample points were first randomly drawn from the various German federal states in accordance with the distribution of the BIK classes on community size. The study subjects were then randomly selected (stratified according to age) from the population registers held in local registry offices. In total, 15,023 children and adolescents (7,538 girls, 7,485 boys) participated in the cross-sectional survey of KiGGS Wave 2; 13,568 of the participants were aged between 3 and 17. The concept and design of KiGGS have been described in detail elsewhere [21-23].

### 2.2 Variables

#### Sports behaviour

This article focuses on sports behaviour, because sports represents a specific type of physical activity that is usually more intense and, therefore, more beneficial to health than general physical activity [24]. Moreover, current data on physical activity from KiGGS Wave 2 has been published in Physical Activity of Children and Adolescents in Germany. Results of the cross-sectional KiGGS Wave 2 study and trends in issue 1/2018 of the Journal of Health Monitoring [10].

KiGGS Wave 2 collected data on sports behaviour of children and adolescents by interviewing 11- to 17-year-old adolescents and the parents of 3- to 10-year-old children. Respondents were asked whether they participated in sports and were told that this referred to all kinds of sports regardless of whether the participation was part of a sports club or not. Physical education at school and exercise in nurseries were not to be accounted for. The question could be answered ‘Yes’ or ‘No’. Respondents who answered ‘Yes’ were also asked how many minutes or hours they usually participated in sports per week. A duration of at least 90 minutes per week and of at least 180 minutes per week were chosen as cut-off points for sports participation, as sports training sessions for children and adolescents usually last 90 minutes.

The parents of 3- to 17-year-old children and adolescents participating in KiGGS Wave 2 were also asked how often they participated in sports themselves. The following answer categories were provided: ‘No sports’, ‘Regularly less than 1 hour per week’, ‘Regularly 1 to 2 hours per week’, ‘Regularly 2 to 4 hours per week’ and ‘Regularly more than 4 hours per week’. In the following analysis, the responses provided to the parents’ question are divided into two categories: ‘Less than 1 hour of sports per week’ and ‘At least 1 hour of sports per week’. Furthermore, the parents were asked about their living environment: ‘Are there any playing or sports facilities in your area of residence that are easily accessible to your child?’ The question specifically asked about playgrounds, sports fields, swimming pools, parks/green spaces. All questions could be answered ‘Yes’, ‘No’ or ‘Don’t know’. The following analyses only consider sports fields, swimming pools and parks/green spaces.

#### Nutrition

KiGGS Wave 2 employed a food frequency questionnaire to assess the consumption of selected food groups [25, 26]. Apart from a number of exceptions in terms of certain foods, this questionnaire is largely comparable with the one used for the KiGGS baseline study.

The respondents were asked which foods they had eaten ‘during the last four weeks’. The 3- to 10-year-olds were asked about a total of 48 food groups, and 11- to 17-year-olds were asked about 53 food groups (these also included alcoholic drinks). Whereas the parents of the 3- to 10-year-olds answered the questionnaire, the 11- to 17-year-olds answered the questions themselves. The questions on consumption frequency followed the pattern: ‘How often did your child/did you eat/drink food/drink X?’ Examples were given in most cases. Each question could be answered with: ‘never’, ‘once per month’, ‘2-3 times per month’, ‘1-2 times per week’, ‘3-4 times per week’, ‘5-6 times per week’, ‘daily’, ‘2 times per day’, ‘3 times per day’, ‘4-5 times per day’, ‘more than five times per day’. ‘More than five times a day’. Data on portion sizes were gathered using questions following the pattern: ‘When your child/you eat/drink food X, how much does your child/do you usually eat/drink?’ Five answer categories were provided in each case, but these varied depending on the type of food in question. They included for instance ‘½ a glass (or less)’, ‘1 glass’, ‘2 glasses’, ‘3 glasses’, ‘4 glasses (or more)’. For some foods, an additional question was asked about the specific way in which the food was consumed (such as to find out the degree to which juices were diluted).

**Figure 1:**
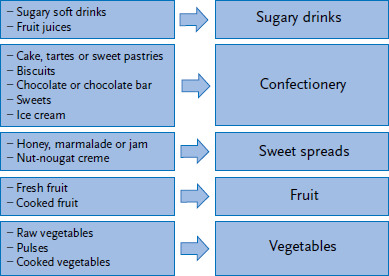
Selected food groups from the food frequency questionnaire used in KiGGS Wave 2 Source: KiGGS Wave 2 (2014-2017)

For the analysis, data on consumption frequencies were converted into the number of occasions a particular food was consumed over a four-week period (28 days). The portions were converted into grams or millilitres. These two values were then multiplied and divided by 28 (consumption frequency × portion size in g/28 days) to obtain an estimated average daily figure. Once these quantities had been calculated, they were then summarised as shown in Figure 1. In addition, in order to compare these data with the ‘5-a-day recommendation’ [27], the portions of fruit and vegetables consumed per day, which may include up to one glass of juice, were summed to provide an estimate of the ‘total amount of fruit and vegetables consumed’.

### 2.3 Statistical analysis

All analyses of sports behaviour were undertaken using the statistics program StataSE14 (Stata Corp., College Station, TX, US, 2015), food consumption analysis were conducted using SAS, version 9.4 (SAS Institute, Cary, NC, US). The complex sampling design resulted in a clustered, stratified sample. In order to improve the study’s representativeness, the analyses were undertaken using weighting. Clustering within the sample was accounted for using statistical procedures for complex samples to provide more valid estimates of variance (including confidence intervals). Prevalence (sports participation) and averages (in the case of nutrition) are provided with 95% confidence intervals for complex samples. Significant differences were assumed in cases where confidence intervals do not overlap.

For the regression analysis (sports participation), individual (age and socioeconomic status; Model 1), interpersonal (parental sports behaviour; Model 2) and environmental factors (sports fields/swimming pools/parks and green spaces; Model 3) were gradually included in the complete case analysis once bivariate relationships had been considered. The models are adjusted for age and socioeconomic status. The way in which socioeconomic status was measured for KiGGS Wave 2 is described in more detail in Socioeconomic status and subjective social status measurement in KiGGS Wave 2, which was published in issue 1/2018 of the Journal of Health Monitoring [28].

## 3. Results

### 3.1 Sports behaviour

This section describes the prevalence of sports behaviour among 3- to 17-year-old girls and boys and its potential influencing factors; these details are also presented in Table 1. 70.9% of 3- to 17-year-old girls and 75.1% of boys of the same age report that they do sports. In this respect, a significant difference was identified between girls and boys. Girls and boys aged between 3 and 17 also differ significantly in the duration of sports participation: 53.9% of girls, but 62.8% of boys, participated in sports for at least 90 minutes per week. Moreover, 31.4% of girls, but 45.0% of boys (aged between 3 and 17), participated in sports for at least 180 minutes per week. No significant gender differences with regard to the factors that could potentially influence sports behaviour were observed: slightly more than half of the mothers and just under half of the fathers of the girls and boys interviewed participated in sports for at least one hour per week. According to their parents, 80% of girls and boys have an easily accessible sports field, park/green space, and about 55% have an easily accessible swimming pool.

The differences linked to gender persist within both age groups (3 to 10 years of age, and 11 to 17 years of age). In both age groups, boys are more likely to report that they participated in sports; however, the difference was only significant among 11- to 17-year-olds. 3- to 10-year-old and 11- to 17-year-old boys participated in sports for at least 90 minutes respectively at least 180 minutes per week more frequently than girls of the same age. No significant gender-related differences were identified in terms of possible influencing factors in either of the age groups.

Older children report more frequently that they participated in sports than younger children. The reported frequency of sports participation for at least 90 minutes and at least 180 minutes per week is higher in older children than in younger ones. Boys between the ages of 11 and 17 report more frequently than boys between 3 and 10 that they participated in sports. Mothers of 11- to 17-year-old girls and boys report more frequently that they participated in sports than mothers of 3- to 10-year-olds. In addition, parents of 11- to 17-year-olds indicate more often than parents of 3- to 10-year-olds that their child has easy access to a sports field, swimming pool or park/green space.

The results of the logistic regression analysis described below are presented in Table 2a and Table 2b where they are arranged according to gender due to the significant differences that were identified between girls and boys. The odds ratios (OR) in the tables indicate the factor by which the odds of sports participation for a selected group is higher or lower compared to the reference category (no sports).

#### Individual factors

There is a positive relationship between sports participation and age as well as between sports participation and socioeconomic status among girls and boys. There is a stronger correlation between sports participation and age among boys than among girls. In contrast, among girls the relationship with an intermediate or high socioeconomic status and sports participation was stronger than among boys. The relationship between individual factors and sports participation remains significant in the multivariate models, but these are weaker than found for the bivariate models.

#### Interpersonal factors

Parental sports participation is associated with children’s sports participation. Girls and boys whose mothers or fathers participated in sports for at least one hour per week are twice as likely to participate in sports than girls and boys whose mothers or fathers participated in sports for less than one hour per week. The relationship remains significant even in the multivariate analyses (Models 2 and 3), but the relationship is weaker in these cases.

#### Environmental factors

There is a positive correlation between an easily accessible sports field and sports participation among girls and boys. The correlation remains significant in the multivariate model even after adjusting for age, socioeconomic status and parental sports participation (Model 3); however, it is weaker in this case. An easily accessible swimming pool as well as an easily accessible park/green space are only associated with sports participation among boys. However, no significant relationship was found when the models were applied in this case.

### 3.2 Nutrition

Table 3 sets out exemplary data about nutrition for the daily intake of certain food groups that contain high levels of sugar (sugary drinks, confectionery and sweet spreads) as well as for other food groups that are indicative of a more healthier lifestyle (water, fruit and vegetables).

#### Sugary drinks

On average, 3- to 17-year-olds drink more than half a litre of sugary drinks per day. However, the figures are significantly lower for girls aged between 3 and 10 (454ml per day) compared to 11- to 17-year-old girls (569ml per day). Nevertheless, the corresponding estimates for boys aged between 3 and 10-years-of-age (568ml per day), and for those aged between 11 and 17 (708ml per day) are significantly higher than for girls. In comparison to the results from the KiGGS baseline study, the average daily consumption of these drinks has fallen by about one quarter; these changes are statistically significant for all of the age and gender groups shown in Table 3.

#### Confectionery

On average, 3- to 17-year-olds consume 68.9 grams of Confectionery per day. 3- to 10-year-old girls are estimated to eat a significantly lower amount (60.6g per day) compared to 11- to 17-year-old girls (73.1g per day). Boys aged between 3 and 10 years of age consume significantly more confectionery (68.4g per day) than girls of the same age. 11- to 17-year-old boys eat 74.1 grams per day, which is almost the same as the amount consumed by girls of this age. The reports from young people demonstrate that they are consuming significantly lower amounts of confectionery compared to the period during which the KiGGS baseline study was conducted. Depending on gender and age group, this rate has fallen as much as 20% to 30% and is statistically significant.

#### Sweet spreads

On average, 3- to 17-year-olds eat 12.2 grams of sweet spreads per day. 3- to 10-year-old girls are estimated to consume significantly less at 10.6 grams per day compared to 11- to 17-year-old girls (12.3g per day). 3- to 10-year-old boys eat about the same amount as girls of the same age (11.3g per day), whereas boys aged between 11 and 17 consume 14.8g per day, which is significantly more than girls of the same age and younger boys. The mean values are similar to those identified by the KiGGS baseline study.

#### Water

3- to 17-year-olds tend to drink an average of almost one and a half litres of water per day. However, girls aged between 3 and 10 drink significantly less (1.246ml per day) than 11- to 17-year-old girls (1.665ml per day). Boys drink a similar amount of water as girls, with boys aged between 3 and 10 drinking 1.273 millilitres per day and 11- to 17-year-olds drinking 1.527 millilitres per day. Compared to the KiGGS baseline study, mean daily water consumption has increased greatly. Depending on age and gender, water consumption has increased significantly by between 50% and 90%.

#### Fruit

On average, 3- to 17-year-olds eat 252 grams of fruit per day. 3- to 10-year-old girls eat a significantly greater amount (286g per day) than 11- to 17-year-old girls (252g per day). 3- to 10-year-old boys consume slightly less than girls of the same age at 267 grams per day. However, 11- to 17-year-old boys eat much less fruit at 199 grams per day than girls of the same age and younger boys; this constitutes a significant difference. Young people aged between 3 and 10 currently eat significantly greater levels of fruit compared to the figures identified by the KiGGS baseline study. Nevertheless, the quantities eaten by older girls and boys have hardly changed.

#### Vegetables

On average, 3- to 17-year-olds consume 125 grams of vegetables per day. 3- to 10-year-old girls eat slightly more (142g per day) than 11- to 17-year-old girls (129g per day), although this difference is not significant. Boys aged between 3 and 10 consume slightly less vegetables (127g per day) than girls of the same age; boys aged between 11 and 17 eat 102 grams per day of vegetables, which is significantly less than girls of the same age and younger boys. Compared with the KiGGS baseline study, the quantities of vegetables consumed by girls and boys aged between 3 and 10 years of age have increased significantly. In contrast, girls and boys aged between 11 and 17 currently eat significantly less vegetables.

#### The number of portions of fruit or vegetables eaten per day

13.2% of 3- to 17-year-olds eat less than one portion of fruit or vegetables per day; 51.3% eat between one and three portions, 21.4% eat between three and five, and 14.1% meet the recommendations made by the ‘5 a day’ campaign and eat five or more portions of fruit and vegetables per day (Table 4). The proportion of young people who eat five or more portions of fruit and vegetables per day is relatively similar across all age groups: 17.2% of 3- to 10-year-old girls, 14.0% of 11- to 17-year-old girls and 15.5% of 3- to 10 year-old boys meet the recommendations. The proportion is only significantly lower among 11- to 17-year-old boys at 9.3%. The proportion of 3- to 10-year-olds that eats five or more portions of fruit and vegetables per day has increased significantly compared to the figures from the KiGGS baseline study. Nevertheless, the proportion of 11- to 17-year-olds that does so has remained roughly the same.

## 4. Discussion

The results provide an overview of selected indicators that can influence the balance between energy intake and energy use, and, thus, can affect the onset or course of obesity.

The results for sports behaviour show that more than 70% of children and adolescents state that they participated in sports; boys do so more often than girls, and older children do so more often than younger children. Moreover, various individual, interpersonal and environmental factors are related to sports behaviour in children and adolescents. Younger age, male gender and a high socioeconomic status are associated with sports participation in childhood and adolescence. Parental sports participation is also positively associated with sports participation among children and adolescents. The living environment – particularly an easily accessible sports field – also seems to have a positive influence on sports participation, regardless of socioeconomic status.

However, when interpreting the results it is important to remember that sports participation in KiGGS Wave 2 refer to all kinds of sports practiced by 3- to 17-year-olds in their leisure time, regardless of whether the participation was part of a sports club or not. Overall, the results presented here on sports behaviour are comparable with those from the Health Behaviour in School-aged Children (HBSC) study [29, 30] and are in line with the statistics collected by the German Olympic Sports Confederation [31]. However, the prevalence related to sport behaviour identified by the HBSC and KiGGS studies cannot be compared exactly as they used different indicators. For example, the HBSC study focused on sports participation that lasted for at least two hours per week (the children were expected to be out of breath or begin to sweat), whereas KiGGS Wave 2 asked whether the children participated in sports (‘Yes’ or ‘No’) and focused on sports participation that lasted for at least 90 minutes per week as well as for at least 180 minutes per week.

Other studies have also demonstrated the relationships identified here between interpersonal factors in the form of parental sports participation [32-34] and between environmental factors and sports participation in children [8, 35]. In terms of environmental factors, the question remains as to whether parents or children who frequently use their local environment for activities may be more likely to answer affirmatively about accessible play and sports facilities (such as a sports field, park/green space and swimming pool) as parents who are rarely or never active (with their child) in the local area. A cross-sectional study of the relationship between subjective and objective data on the local environment and sports behaviour found that the subjective parental assessment of the local environment is more strongly associated with their sports participation than the objective results about the local area [36]. As such, the issue of the local environment also depends on whether the parks, green spaces, swimming pools and sports fields that are viewed as easily accessible are actually used for sports activities, as well as the distance to these facilities, and on the parents’ opinions on the quality of footpaths, cycle lanes and road safety [38].

A comparison of trends in terms of sports behaviour between the KiGGS baseline study (2003-2006) and KiGGS Wave 2 (2014-2017) is problematic as changes were made to the method used to query information. Within the concept of physical activity, a slight decline was identified among 3- to 10-year-old girls in terms of moderate to vigorous physical activity that lasted for at least 60 minutes per day between KiGGS Wave 1 (2009-2012) and KiGGS Wave 2 (2014-2017) [10]. Between 2002 and 2014, the results of the HBSC study of 11-, 13- and 15-year-olds show that physical activity has increased, and this includes at least 60 minutes of moderate to vigorous physical activity per day. However, the level is significantly lower among 15-year-old girls than for boys of this age and children in other age groups [39]. The HBSC study also shows an increase in very intense physical activity undertaken at least four times a week among both girls and boys [39]. The Motorik-Module (MoMo), which gathers data using in-depth questions about physical activity and sports from a sub-sample of the KiGGS study, also identified a decline in sports activity conducted outside a sports club and an increase in sports activity undertaken within sports clubs between the MoMo baseline study (2003-2006) and MoMo Wave 1 (2009-2012) [40].

Several food groups were selected for evaluation using the data collected on food consumption for KiGGS Wave 2. These foods are at the focus of health policy debates in terms of the prevention of obesity. On the one hand, they include sugary drinks, confectionery and sweet spreads, all of which are conducive to the development of obesity when consumed in high quantities; on the other, they include water, fruit and vegetables, which are associated with reduced obesity. The results of the indicators on dietary behaviour suggest that younger children and girls drink fewer sugary drinks and eat less confectionery and sweet spreads, as well as greater amounts of fruit and vegetables than older children and boys. However, the results point to different trends. Whereas the consumption of confectionery and sugary drinks has declined significantly among 3- to 17-year-olds since the KiGGS baseline study was conducted, 11- to 17-year-olds, in particular, now eat significantly less vegetables compared to about ten years ago. The proportion of girls and boys who meet the recommendation of the German Nutrition Society to eat at least five portions of fruit and vegetables per day has increased significantly among 3- to 10-year-olds over the last ten years, but the proportion of those who reach this recommendation remains very low at 14%.

When interpreting the results, it is important to consider that the results from the food questionnaire can only provide rough descriptions of the foods that were eaten compared to data collected by more extensive surveys of nutrition, and, therefore, that the estimated quantities only represent rough indicators of patterns of consumption. For example, chocolate drinks and other mixed milk drinks also contain sugar, but questions about them were posed as part of the food group ‘milk’. As such, these drinks are not displayed separately in the tables and are not considered here. In addition, although the market share of flavoured water is currently relatively small [41], some of these products contain a substantial amount of sugar. The respondents probably also classified these drinks as water when answering the questionnaire.

Since the ways in which data was collected about food by the KiGGS baseline study and KiGGS Wave 2 were very similar, results can be presented on developments over time. The presentation of the findings focuses on averages, as this simplifies comparison with other sets of results. However, this does not always do justice to the spread of the data. The average consumption of water in KiGGS Wave 2, for example, is relatively high, partly because a large group of children and adolescents reported very high values. The median amounts of water that the respondents drink are significantly lower, but this also applies to the KiGGS baseline study. The increase in water consumption coincides with an increase in per capita consumption of mineral water, which has increased from 138.1 litres in 2008 to 151.9 litres in 2015 [42]. The decrease in per capita consumption of fizzy drinks (82.9 litres in 2012, 78.2 litres in 2016 [41]) and fruit juices (37.4 litres in 2008, 33.0 litres in 2015 [42]) also fits well with the reduction in the consumption of sugary drinks observed in KiGGS. However, these figures refer to the entire population and may not necessarily match the trends in consumption among children and adolescents. Despite this, the HBSC study of 11-, 13- and 15-year-olds in Germany identified a reduction in the daily consumption of soft drinks between 2002 and 2014 [43]. The figures for chocolates, cocoa-containing spreads, confectionery and pastry products have hardly changed between 2007 and 2014. The per capita consumption of vegetables has increased from 86.4kg per year in 2005/2006 to 98.6kg per year in 2014/2015. However, it has fallen for fruit from 78.6kg per year in 2005/2006 to 66.5kg per year in 2014/2015 [42]. Therefore, the findings only coincide with the results of KiGGS Wave 2 with regard to vegetable consumption by 3- to 10-year-olds.

The levels estimated for 3- to 10-year-olds are lower than those for 11- to 17-year-olds due to the lower energy and nutritional needs of younger children. It is striking however, that the estimated daily amount of fruit eaten by 3- to 10-year-olds is slightly higher than the level estimated for 11- to 17-year-olds. This could be due to the fact that the parents of 3- to 10-year-olds completed the questionnaire and may have been more likely to provide socially desirable responses. It could also be due to the fact that consumption actually decreases during adolescence.

Within the objective ‘Grow up healthy’ of the German national health targets (www.gesundheitsziele.de) there is a focus on life skills, exercise and nutrition [44]. Patterns of behaviour develop early in life and are difficult to change. As such, parents acting as role models, behaviour in the family and peer group as well as in the living environment (settings) in which children and adolescents grow up can have a very early positive effect on health-related behaviour. Physically active children often become physically active adults [45]. Dietary habits have also been observed to persist into adulthood [46-47]. In addition to individual behaviour, research is increasingly focusing on the importance and consideration of issues such as environmental factors on physical activity, diet and obesity [48]. They also need to be taken into account as an integral part of the implementation of policy measures. Legislative approaches, such as providing better access to healthy food (for example, through tax reforms) and designing settings which promote good health especially for socially disadvantaged children (such as through the physical activity friendly design of areas where socially disadvantaged people live), are also required [1]. As part of an early approach to intervention, healthy eating, physical activity and sports need to be anchored in school curricula and local environments, and opportunities need to be developed that encourage children to eat healthier foods and be active every day [49]. This means that teachers and educators also need to receive support when implementing activities that promote good health and parents also require help to motivate their children to become more active, less sedentary and to eat a balanced diet. A combination of measures, therefore, needs to be put in place if children and adolescents are to meet the national recommendations on physical activity and healthy eating.

## Key statements

- More than 70% of 3- to 17-year-olds do sports.
- Parents’ sports participation and an activity-friendly living environment are associated with children’s and adolescents’ sports participation.
- Fewer sugary drinks and more water are consumed than ten years ago.
- Girls have healthier diets than boys, and 3- to 10-year-olds eat healthier than 11- to 17-year-olds.
- 11- to 17-year-olds currently eat fewer vegetables than they did ten years ago.

## Figures and Tables

**Table 1:** Prevalences of sports participation and potential influencing factors for 3- to 17-year-olds according to gender and age (n=6,565 girls, n=6,413 boys) Source: KiGGS Wave 2 (2014-2017)

Total			Age group			
			3-10 years		11-17 years	
	%	(95% CI)	%	(95% CI)	%	(95% CI)
**Girls**						
Sports participation (yes or no)	70.9	(69.3-72.5)	69.9	(67.5-72.2)	72.1	(69.7-74.4)
Sports ≥ 90 minutes per week	53.9	(52.2-55.7)	48.2	(45.9-50.5)	60.3	(57.6-62.8)
Sports ≥180 minutes per week	31.4	(29.9-33.0)	25.4	(23.3-27.5)	38.1	(35.8-40.5)
**Potential influencing factors**						
Mother: ≥1 hour of sport per week	50.6	(48.9-52.4)	46.8	(44.4-49.2)	54.8	(52.2-57.3)
Father: ≥1 hour of sport per week	47.3	(45.5-49.1)	46.6	(44.1-49.2)	48.1	(45.6-50.7)
Sports ground nearby	80.4	(78.4-82.3)	75.6	(73.1-77.9)	85.3	(82.9-87.4)
Swimming pool nearby	55.1	(51.2-58.9)	47.6	(43.5-51.8)	62.9	(58.7-67.0)
Park/green space nearby	80.9	(78.7-83.0)	77.5	(74.7-80.0)	84.6	(82.2-86.8)
**Boys**						
Sports participation (yes or no)	75.1	(73.5-76.6)	70.4	(68.0-72.7)	80.3	(78.2-82.3)
Sports ≥ 90 minutes per week	62.8	(61.0-64.6)	53.7	(51.4-55.9)	73.1	(70.8-75.3)
Sports ≥180 minutes per week	45.0	(43.2-46.7)	34.5	(32.5-36.6)	56.7	(54.2-59.2)
**Potential influencing factors**						
Mother: ≥1 hour of sport per week	50.3	(48.4-52.1)	47.3	(44.7-49.8)	53.4	(50.6-56.3)
Father: ≥1 hour of sport per week	49.2	(47.3-51.1)	48.1	(45.8-50.5)	50.4	(47.7-53.0)
Sports ground nearby	83.8	(81.9-85.5)	79.3	(77.0-81.6)	88.3	(86.2-90.1)
Swimming pool nearby	56.3	(52.2-60.2)	49.8	(45.5-54.1)	62.9	(58.5-67.2)
Park/green space nearby	79.7	(77.5-81.8)	77.0	(74.3-79.5)	82.6	(80.1-84.8)

CI=confidence interval

**Table 2a:** Step-by-step analysis of the relationships between individual, interpersonal, and environmental influencing factors and sports participation among 3- to 17-year-old girls (n=5,431) Source: KiGGS Wave 2 (2014-2017)

	Girls’ sports participation							
	Bivariate relationships		Model 1 (age, social status)		Model 2 (Model 1 + parental sports)		Model 3 (Model 2 + environmental factors)	
	OR	(95% CI)	OR	(95% CI)	OR	(95% CI)	OR	(95% CI)
**Age group**								
3-6 years	1.00		1.00		1.00		1.00	
7-10 years	2.62	(2.09-3.29)	2.71	(2.14-3.43)	2.64	(2.08-3.36)	2.72	(2.13-3.47)
11-13 years	2.55	(1.98-3.29)	2.70	(2.08-3.49)	2.56	(1.97-3.34)	2.58	(1.95-3.40)
14-17 years	1.33	(1.08-1.63)	1.50	(1.22-1.86)	1.37	(1.09-1.72)	1.39	(1.11-1.75)
**Socioeconomic status**								
Low	1.00		1.00		1.00		1.00	
Medium	2.25	(1.76-2.87)	2.29	(1.79-2.91)	1.96	(1.51-2.54)	1.91	(1.46-2.49)
High	4.13	(3.06-5.58)	4.26	(3.14-5.78)	3.08	(2.23-4.26)	3.11	(2.24-4.31)
**Sports participation of the mother**								
<1 hour per week	1.00				1.00		1.00	
≥1 hour per week	2.48	(2.10-2.93)			1.92	(1.60-2.31)	1.89	(1.57-2.28)
**Sports participation of the father**								
<1 hour per week	1.00				1.00		1.00	
≥1 hour per week	2.01	(1.70-2.37)			1.42	(1.16-1.73)	1.41	(1.14-1.74)
**Sports ground nearby**								
No	1.00						1.00	
Yes	1.47	(1.20-1.80)					1.36	(1.08-1.70)
**Swimming pool nearby**								
No	1.00						1.00	
Yes	1.14	(0.96-1.35)					0.97	(0.80-1.18)
**Park/green space nearby**								
No	1.00						1.00	
Yes	0.97	(0.79-1.20)					0.79	(0.62-1.00)

OR = odds ratio, CI=confidence interval

**Table 2b:** Step-by-step analysis of the relationships between individual, interpersonal, and environmental influencing factors and sports participation among 3- to 17-year-old boys (n=5,470) Source: KiGGS Wave 2 (2014-2017)

	Boys’ sports participation							
		Bivariate relationships		Model 1 (age, social status)		Model 2 (Model 1 + parental sports)		Model 3 (Model 2 + environmental factors)	
	OR	(95% CI)	OR	(95% CI)	OR	(95% CI)	OR	(95% CI)
**Age group**								
3-6 years	1.00		1.00		1.00		1.00	
7-10 years	3.37	(2.67-4.24)	3.62	(2.88-4.56)	3.61	(2.86-4.56)	3.49	(2.76-4.41)
11-13 years	3.47	(2.62-4.61)	3.66	(2.74-4.89)	3.59	(2.67-4.82)	3.28	(2.43-4.44)
14-17 years	2.40	(1.89-3.05)	2.64	(2.07-3.36)	2.47	(1.95-3.14)	2.22	(1.74-2.84)
**Socioeconomic status**								
Low	1.00		1.00		1.00		1.00	
Medium	1.77	(1.39-2.26)	1.94	(1.50-2.52)	1.69	(1.31-2.19)	1.65	(1.26-2.15)
High	2.87	(2.10-3.91)	3.33	(2.44-4.55)	2.49	(1.82-3.41)	2.44	(1.79-3.32)
**Sports participation of the mother**								
<1 hour per week	1.00				1.00		1.00	
≥1 hour per week	2.25	(1.88-2.68)			1.64	(1.36-1.98)	1.55	(1.28-1.87)
**Sports participation of the father**								
<1 hour per week	1.00				1.00		1.00	
≥1 hour per week	2.15	(1.79-2.57)			1.70	(1.39-2.08)	1.69	(1.39-2.06)
**Sports ground nearby**								
No	1.00						1.00	
Yes	2.08	(1.66-2.59)					1.57	(1.21-2.04)
**Swimming pool nearby**								
No	1.00						1.00	
Yes	1.40	(1.17-1.68)					1.16	(0.95-1.42)
**Park/green space nearby**								
No	1.00						1.00	
Yes	1.40	(1.14-1.72)					0.99	(0.80-1.22)

OR = odds ratio; CI=confidence interval

**Table 3:** Mean values for daily amounts of foods with 95% confidence intervals for 3- to 17-year-olds according to gender and age (KiGGS baseline study n=6,918 girls, n=7,186 boys; KiGGS Wave 2 n=6,568 girls, n=6,466 boys) Source: KiGGS baseline study (2003-2006), KiGGS Wave 2 (2014-2017)

Gender/age group Indicator (unit of measure)	KiGGS baseline study		KiGGS Wave 2	
	AV	95% CI	AV	95% CI
**Girls**				
Sugary drinks (ml)	704.8	(670.4-739.1)	508.7	(469.4-548.1)
Confectionery (g)	85.2	(80.7-89.7)	66.6	(62.8-70.3)
Sweet spreads (g)	10.3	(9.9-10.8)	11.4	(10.8-11.9)
Drinking water (ml)	874.5	(830.0-918.9)	1,445.1	(1,390.0-1,500.2)
Fruit (g)	242.4	(231.2-253.5)	269.8	(259.1-280.6)
Vegetables (g)	129.8	(125.3-134.2)	135.7	(129.7-141.6)
**Boys**				
Sugary drinks (ml)	843.4	(804.1-882.7)	634.1	(592.1-676.1)
Confectionery (g)	95.2	(91.2-99.2)	71.1	(67.8-74.4)
Sweet spreads (g)	12.3	(11.8-12.9)	12.9	(12.2-13.6)
Drinking water (ml)	816.1	(776.0-856.3)	1,392.4	(1,331.0-1,453.8)
Fruit (g)	203.9	(195.2-212.6)	234.9	(224.6-245.3)
Vegetables (g)	119.7	(116.1-123.4)	115.2	(110.7-119.6)
**Total**				
Sugary drinks (ml)	775.7	(746.4-805.1)	573.0	(539.5-606.4)
Confectionery (g)	90.3	(86.7-93.9)	68.9	(65.9-71.9)
Sweet spreads (g)	11.3	(10.9-11.7)	12.2	(11.7-12.6)
Drinking water (ml)	844.6	(810.4-878.7)	1,418.1	(1,369.7-1,466.5)
Fruit (g)	222.7	(214.9-230.4)	252.0	(243.8-260.1)
Vegetables (g)	124.6	(121.7-127.6)	125.2	(121.1-129.2)
**Girls, 3-10 years**				
Sugary drinks (ml)	626.1	(585.6-666.7)	454.0	(405.4-502.7)
Confectionery (g)	75.4	(70.7-80.1)	60.6	(57.3-63.9)
Sweet spreads (g)	9.9	(9.3-10.5)	10.6	(9.9-11.2)
Drinking water (ml)	649.2	(601.0-697.3)	1,246.2	(1,173.7-1,318.8)
Fruit (g)	234.7	(223.0-246.4)	286.0	(273.4-298.6)
Vegetables (g)	115.0	(110.0-120.0)	142.2	(133.1-151.3)
**Girls, 11-17 years**				
Sugary drinks (ml)	780.1	(731.0-829.2)	569.2	(511.5-627.0)
Confectionery (g)	94.6	(88.3-100.8)	73.1	(66.4-79.9)
Sweet spreads (g)	10.7	(10.1-11.3)	12.3	(11.3-13.2)
Drinking water (ml)	1,089.8	(1,025.5-1,154.2)	1,665.4	(1,583.3-1,747.4)
Fruit (g)	249.7	(233.4-266.1)	251.9	(236.0-267.9)
Vegetables (g)	143.9	(137.1-150.7)	128.5	(121.7-135.3)
**Boys, 3-10 years**				
Sugary drinks (ml)	722.1	(679.0-765.2)	568.1	(515.1-621.1)
Confectionery (g)	82.3	(78.0-86.5)	68.4	(64.0-72.9)
Sweet spreads (g)	10.7	(10.1-11.4)	11.3	(10.6-11.9)
Drinking water (ml)	671.5	(625.6-717.3)	1,272.9	(1,193.5-1,352.3)
Fruit (g)	216.1	(206.8-225.4)	267.1	(253.0-281.3)
Vegetables (g)	109.7	(104.6-114.8)	127.4	(121.1-133.7)
**Boys, 11-17 years**				
Sugary drinks (ml)	962.4	(907.0-1,017.7)	708.0	(648.2-767.9)
Confectionery (g)	107.9	(102.2-113.6)	74.1	(69.1-79.0)
Sweet spreads (g)	13.9	(13.1-14.6)	14.8	(13.5-16.1)
Drinking water (ml)	957.9	(900.5-1,015.3)	1,526.8	(1,441.8-1,611.8)
Fruit (g)	191.9	(179.1-204.7)	198.8	(186.2-211.3)
Vegetables (g)	129.5	(124.0-135.1)	101.5	(95.3-107.6 )

AV=average, CI=confidence interval, ml=millilitre, g=gram

**Table 4:** Mean values for daily portions of fruit and vegetables as a percentage with 95% confidence intervals for 3- to 17-year-olds according to gender and age (KiGGS baseline study n=6,918 girls, n=7,186 boys; KiGGS Wave 2 n=6,568 girls, n=6,466 boys) Source: KiGGS baseline study (2003-2006), KiGGS Wave 2 (2014-2017)

Gender/ age group	KiGGS baseline study		KiGGS Wave 2	
	%	(95% CI)	%	(95% CI)
**Girls, 3-17 years**				
<1 Portion	9.9	(8.9-10.9)	11.2	(10.2-12.3)
1-<3 Portionen	53.5	(51.7-55.4)	50.9	(49.3-52.5)
3-<5 Portionen	22.9	(21.6-24.2)	22.2	(21.0-23.6)
≥5 Portionen	13.7	(12.7-14.9)	15.7	(14.6-16.8)
**Boys, 3-17 years**				
<1 Portion	12.8	(11.8-13.8)	15.1	(13.9-16.4)
1-<3 Portionen	55.1	(53.5-56.6)	51.7	(50.2-53.3)
3-<5 Portionen	21.3	(20.2-22.5)	20.6	(19.3-21.9)
≥5 Portionen	10.9	(10.0-11.9)	12.6	(11.4-13.8)
**Total, 3-17 years**				
<1 Portion	11.3	(10.6-12.1)	13.2	(12.4-14.0)
1-<3 Portionen	54.3	(53.1-55.6)	51.3	(50.3-52.4)
3-<5 Portionen	22.1	(21.2-23.0)	21.4	(20.4-22.4)
≥5 Portionen	12.3	(11.5-13.1)	14.1	(13.3-15.0)
**Girls, 3-10 years**				
<1 Portion	8.4	(7.3-9.6)	7.3	(6.3-8.5)
1-<3 Portionen	57.0	(54.9-59.0)	50.7	(48.5-52.8)
3-<5 Portionen	22.8	(21.0-24.6)	24.9	(23.0-26.8)
≥5 Portionen	11.9	(10.7-13.2)	17.2	(15.6-19.0)
**Girls, 11-17 years**				
<1 Portion	11.3	(9.9-12.8)	15.5	(13.8-17.4)
1-<3 Portionen	50.3	(47.8-52.8)	51.2	(48.7-53.7)
3-<5 Portionen	23.0	(21.2-24.8)	19.3	(17.5-21.3)
≥5 Portionen	15.5	(14.0-17.1)	14.0	(12.4-15.7)
**Boys, 3-10 years**				
<1 Portion	9.3	(8.1-10.6)	8.5	(7.3-10.0)
1-<3 Portionen	57.0	(55.0-58.9)	51.3	(49.1-53.5)
3-<5 Portionen	21.9	(20.3-23.6)	24.6	(22.7-26.7)
≥5 Portionen	11.8	(10.6-13.2)	15.5	(14.0-17.2)
**Boys, 11-17 years**				
<1 Portion	16.2	(14.7-17.7)	22.5	(20.5-24.6)
1-<3 Portionen	53.2	(51.1-55.4)	52.2	(49.9-54.6)
3-<5 Portionen	20.7	(19.0-22.5)	16.0	(14.4-17.8)
≥5 Portionen	9.9	(8.8-11.2)	9.3	(7.9-10.9)

CI=confidence interval
